# Patients’ perspective on allergen immunotherapy for respiratory allergy

**DOI:** 10.1097/ACI.0000000000001110

**Published:** 2025-09-23

**Authors:** Francesco Catamerò, Simona Barbaglia, Enrico Heffler, Mattia Giovannini, Giovanni Paoletti

**Affiliations:** a Allergy Unit, Meyer Children's Hospital IRCCS; b Department of Health Sciences, University of Florence, Florence; c Associazione Nazionale pazienti “Respiriamo Insieme-APS”, Padua; d Department of Biomedical Science, Humanitas University, Pieve Emanuele (Milano); e Personalized Medicine, Asthma and Allergy, IRCCS Humanitas Research Hospital, Rozzano, Italy

**Keywords:** adherence, allergen Immunotherapy, patient's association, patient's perspective

## Abstract

**Purpose of review:**

This review explores patients’ perspective on allergen immunotherapy (AIT) for respiratory allergy, addressing awareness, motivations, adherence challenges, perceived benefits and risks, and the importance of education and shared decision-making. It also summarizes the data on patient-reported outcomes, considers the role of patient associations, and outlines future directions for enhancing adherence and advancing patient-centered care.

**Recent findings:**

AIT is the only treatment capable of modifying the natural course of allergic diseases, providing lasting benefits in terms of symptom reduction, quality of life (QoL), and asthma control. Despite its efficacy and safety, AIT remains underused due to several factors, including cost, misinformation, patient skepticism, and adherence challenges. Limited reimbursements further restrict access.

**Summary:**

The patient's perspective is crucial in AIT, as it directly impacts adherence and treatment outcomes. Allergic rhinitis and asthma significantly reduce the QoL, especially when poorly controlled, but their burdens are often underestimated. Adherence to AIT depends on multiple factors including age, physician engagement, perceived efficacy, convenience, education, and socioeconomic status. Effective communication, shared decision-making, and tailored education enhance long-term compliance, while financial barriers and lack of reimbursement remain significant obstacles. Patient-reported outcome measures (PROMs) are essential tools for assessing symptom burden, disease control, and QoL, supporting clinical decisions and research. Validated PROMs, as well as combined symptom-medication scores, help personalize care and are increasingly integrated into digital platforms for real-time monitoring. Respiratory patient associations play a vital role in promoting education, empowerment, and advocacy, enhancing adherence and access to care.

## INTRODUCTION

In recent decades, allergic diseases have become a major global health concern, affecting up to 20–30% of adults and 40% of children, with an increasing prevalence trend [1–4]. Allergic rhinitis with or without conjunctivitis (AR/C) significantly affects sleep quality; occupational, academic, and school performance; as well as leisure activities; and is frequently associated with asthma [5^▪^,^6^,^7^,8^▪^,^9^]. While allergen avoidance is advised when possible, it is rarely feasible in practice [10]. Consequently, most patients depend on symptomatic pharmacotherapy, which, despite alleviating clinical manifestations, neither modifies the natural history of allergic diseases nor prevents their progression [6,7,9–11].

Allergen immunotherapy (AIT) with subcutaneous (SCIT) or sublingual (SLIT) administration of the culprit allergen(s) is recognized as the only therapeutic approach capable of modifying the natural history of the disease [10,12]. AIT is safe for both children and adults, and its benefits are well established, including a significant reduction in allergic symptoms, improvement in the quality of life (QoL), positive effects on asthma, and potential long-term benefits in preventing its onset or achieving remission [11,13–16]. A treatment duration of at least 3 years is generally required to achieve sustained long-term benefits [17^▪▪^].

Despite its proven efficacy, AIT remains underused due to a range of factors, including cost, misinformation regarding safety, patient skepticism, the nature of physician-patient communication, and disease severity [18,19]. Moreover, adherence remains a significant challenge in both clinical trials and real-world settings [20–22]. SCIT requires frequent clinic visits, posing logistical burdens, while SLIT demands long-term daily commitment, often leading to reduced compliance. Suboptimal adherence ultimately undermines the treatment efficacy and leads to avoidable healthcare costs [23–25]. Additionally, from an economic perspective, AIT reduces the need for symptomatic medications and medical visits, offering clear cost advantages. Still, AIT is reimbursed in only 56% of European countries, with full reimbursement available in just 32% [19,23,26].

In this context, a crucial aspect, often underestimated, to improve both adherence and AIT prescriptions is the careful assessment of the patient's perspective regarding AIT, including their baseline knowledge of the treatment, motivation to initiate therapy, and other factors, such as perceived risks, concerns, and expectations about the potential benefits. This, combined with an understanding of AIT utilization patterns, may reveal information about why it is underutilized, thereby providing valuable insights into how to expand its positive impact on the population.

This review aimed to examine the patient's perspective on AIT for respiratory allergy, focusing on awareness, motivations, perceived benefits and risks, adherence challenges, patient preferences, the role of education, and shared decision-making, and summarizes available data on patient-reported outcomes. Additionally, this review highlights the potential contribution of patient associations and outlines future directions for improving adherence and promoting patient-centered care in AIT. 

**Box 1:**
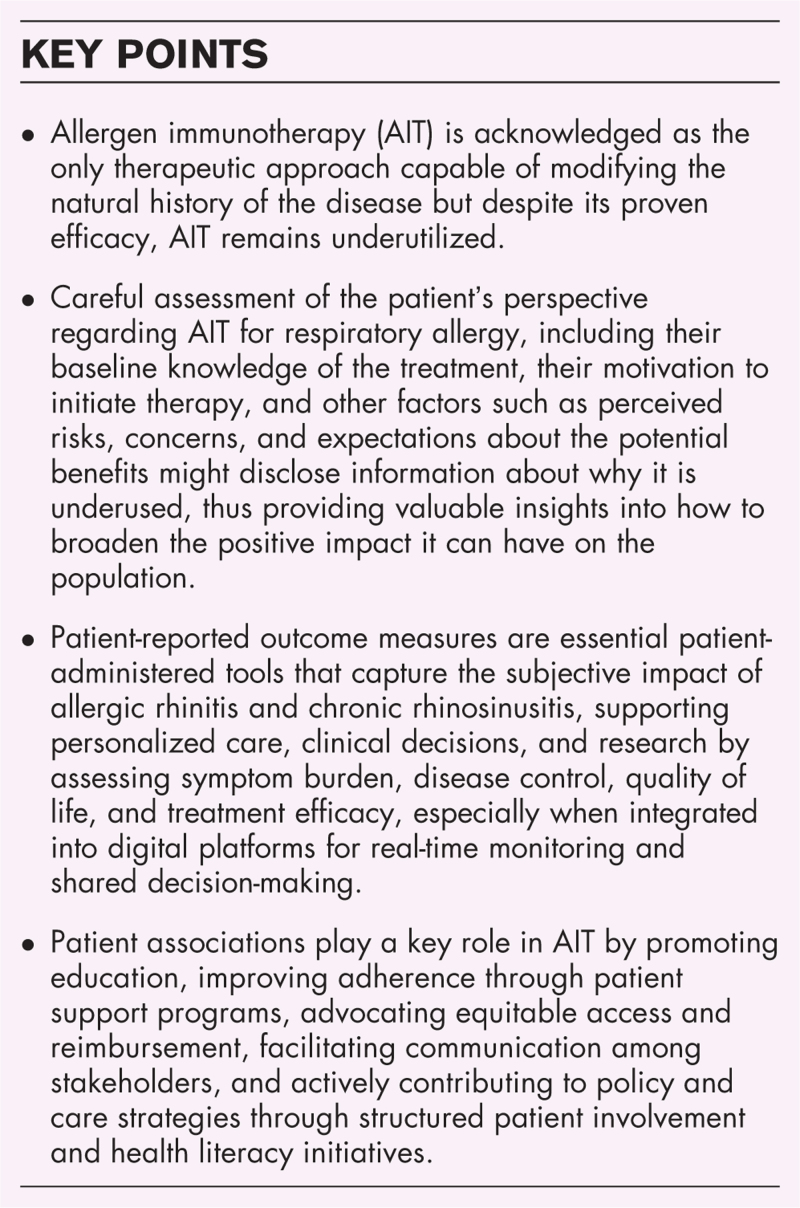
no caption available

## HISTORY AND EVOLUTION OF ALLERGEN IMMUNOTHERAPY

In 1911, Leonard Noon first demonstrated that repeated injections of crude grass pollen extract reduced immediate allergic sensitivity in individuals with hay fever [27]. Shortly after, Freeman observed that these patients experienced fewer respiratory symptoms during the following pollen season [28]. Although the underlying rationale was unclear, the identification of grass pollen as the causative agent of hay fever likely guided this approach, paralleling the early use of vaccines for infectious diseases. In 1954, the first double-blind trial confirmed the efficacy of subcutaneous grass pollen injections for seasonal asthma, identifying high-molecular-weight proteins as the active component [29]. Subsequent studies validated the use of multiallergen mixtures; in 1978, it was shown that AIT effects were allergen-specific, providing benefits only for the targeted allergen season [30]. Despite these milestones, the administration method of subcutaneous immunotherapy has remained largely unchanged for over a century. A 1998 World Health Organization position paper highlighted the efficacy of sublingual immunotherapy but also its risks, especially in poorly controlled asthma, and recognized its promise as a safer alternative [31]. In 1999, it was demonstrated that 3 years of continuous SCIT with grass pollen could offer long-lasting clinical benefits, persisting for years after discontinuation [32]. Subsequently, various randomized controlled trials have confirmed both safety and long-term clinical efficacy, also after discontinuation [17^▪▪^,^33^–^37^].

## MECHANISMS OF ALLERGEN IMMUNOTHERAPY FOR ALLERGY TO INHALANT ALLERGENS

The immunological basis of AIT hinges on its capacity to modulate the dysregulated immune responses characteristic of allergic diseases, shifting them from a pathogenic, T helper (Th) 2-skewed profile toward immune tolerance [17^▪▪^]. In individuals with atopic allergy, environmental allergens elicit immunoglobulin (Ig) E-mediated mast cell activation and eosinophilic inflammation, orchestrated by Th2 cytokines, such as interleukin (IL)-4, IL-5, and IL-13 [38,39]. Historical landmark studies have demonstrated that both hypersensitivity and protective immunity can be passively transferred via the serum, later identified as allergen-specific IgE and IgG/IgA, respectively [40–42]. AIT induces peripheral tolerogenic dendritic cells and promotes the expansion of regulatory T cells (Treg), expressing IL-10, transforming growth factor-β (TGFβ), and IL-35, and thymus-derived FOXP3+ Treg cells [43–48]. Tregs suppress Th2 differentiation through cytokine-mediated and contact-dependent mechanisms [49]. IL-10 induces B cells to undergo isotype class switch toward allergen-specific IgG1, IgG, and TGFβ induces preferential switching of B cells to produce IgA [50–52]. Notably, IgG4 antibodies act as “blocking antibodies,” preventing allergen–IgE complex formation, mast cell and basophil activation, and IgE-facilitated allergen presentation [53]. AIT also modulates innate lymphoid cells 2 (ILC2s), reducing their pro-inflammatory profile and promoting IL-10-expressing regulatory ILC2 subsets [54,55]. Furthermore, AIT increased the differentiation of T follicular regulatory cells-T follicular helper cells (TFH, CXCR5+ Foxp3+) and B regulatory cells. TFH synthesizes the IL-21 and IL-4 necessary for B cell proliferation and antibody synthesis, while B cells secrete IL-10/TGF-β, both of which help with immune modulation [56–58].

Notably, some effects depend on the route of administration of AIT. SCIT induces rapid increases in IgG4 and Treg activity within weeks, whereas SLIT elicits similar effects over a more protracted course, with prominent involvement of oral Langerhans cells and toll-like receptor 4-mediated pathways [59–64]. Additionally, epicutaneous immunotherapy (EPIT), a recently developed route of administration, induces robust T cell-mediated responses, albeit with modest IgG4 production, compared with SCIT and SLIT [65,66]. Additionally, intralymphatic immunotherapy (ILIT), another recently described route of administration, seems to induce both IgG2 and IgG4 and increased IL-10, IFN-γ, and IL-4 levels but in a shorter time than SCIT [67].

## ROUTES OF ADMINISTRATION

Over the years, various routes for AIT administration have been explored. SCIT remains the most widely adopted approach, with robust evidence supporting its long-term efficacy in AR/C [68]. SCIT involves an initial updosing phase with weekly injections followed by monthly maintenance doses for a minimum of 3 years. Its implementation is often limited by the need for frequent physician-supervised visits over a prolonged 3–5-year period and the potential for adverse effects, ranging from local injection site reactions to, albeit rarely, anaphylaxis [24,69].

SLIT represents the second most extensively studied route of AIT administration. The oral mucosa, recognized as an immunologically privileged site, facilitates daily exposure to allergens with a lower risk of systemic hypersensitivity [70,71]. SLIT is self-administered via daily tablets or drops placed under the tongue, typically following an initial 30-min supervised dose in a clinical setting [72]. It offers a favorable safety profile, compared with SCIT, with a significantly reduced risk of severe adverse reactions [73]. Robust evidence from meta-analyses and randomized controlled trials supports the efficacy of SLIT in both seasonal (e.g., grass, ragweed, birch, and Japanese cedar) and perennial (e.g., house dust mites) AR [74–79]. Moreover, long-term benefits have been demonstrated in adults and children following 3 years of treatment, with sustained clinical effects persisting for at least 2 years post discontinuation [33,80–82]. Additionally, SLIT has demonstrated clinical efficacy in house dust mite–induced allergic asthma, reducing both inhaled corticosteroid requirements and asthma exacerbation rates [83,84]. A secondary preventive role of immunotherapy in asthma was suggested by studies of both SLIT and SCIT in children aged 5–12 years with seasonal rhinitis, in whom 3 years of treatment reduced both the symptoms of asthma and requirement for asthma medication at 5 years [35,82].

EPIT represents an emerging route of AIT delivery that involves transdermal administration of allergens via skin patches applied after tape stripping. This technique enhances antigen penetration by disrupting the stratum corneum and activating the keratinocyte-mediated cytokine release [85]. In a randomized, double-blind, placebo-controlled trial using grass pollen allergens, EPIT demonstrated higher patient-reported treatment satisfaction, compared with placebo, despite no significant differences in nasal provocation tests or rescue medication use [86]. Mild local reactions, such as eczematous lesions at the patch site, were the most common adverse events, with no severe systemic reactions reported [86,87]. Another dose-ranging trial confirmed a positive dose–response relationship, with the highest dose group showing over 30% symptom reduction in the first season and 24% in the subsequent season. However, increased efficacy was associated with a higher incidence of adverse events, including systemic reactions in a minority of patients, leading to treatment discontinuation [88]. In the context of food allergy, EPIT has emerged as a promising therapeutic approach. In children and toddlers with peanut allergy, transdermal delivery of peanut extract via skin patches has shown promising results in desensitizing children to peanuts and increasing the peanut dose that triggered allergic symptoms. The adverse events were predominantly mild and localized to the application site, supporting EPIT's favorable safety profile [89^▪▪^,^90^].

ILIT involves the ultrasound-guided injection of allergen extracts into lymph nodes, typically in the groin [91]. Small placebo-controlled trials have demonstrated modest efficacy against grass and tree pollen allergies [92,93]. Notably, a study using a recombinant Fel d 1 fusion protein in cat-allergic individuals showed protection against nasal challenge after three ILIT injections [94]. While several trials have confirmed the efficacy of short-course ILIT for grass pollen allergy, the findings remain inconsistent across studies and further large-scale trials are warranted [91–95].

## PATIENTS’ PERSPECTIVE ON ALLERGEN IMMUNOTHERAPY

**FIGURE 1:**
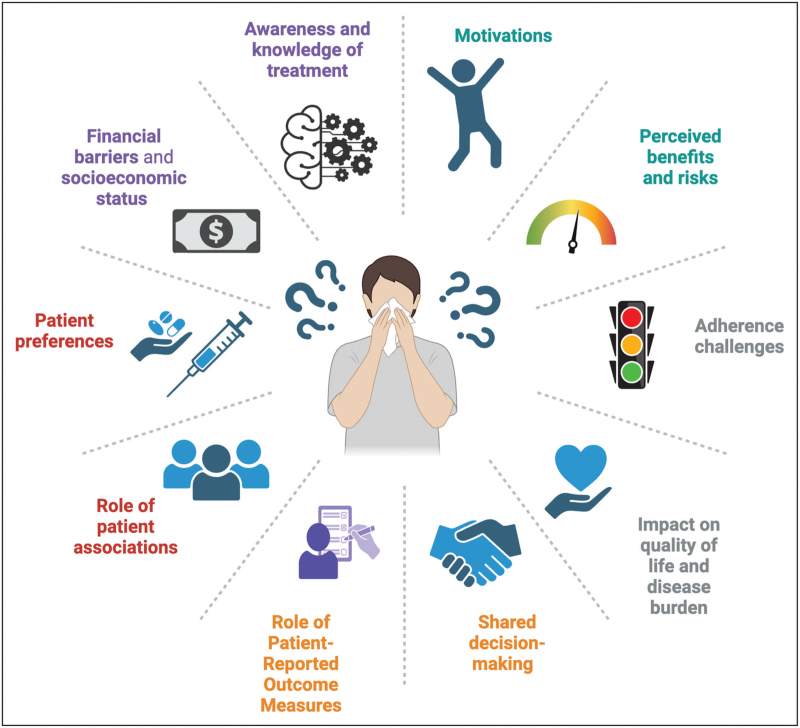
Clinical determinants shaping patients' perspective in allergen immunotherapy: implications for personalized care. Created with BioRender.com.

A crucial aspect in the context of AIT is the patients’ perspective, encompassing how individuals perceive the treatment and how this viewpoint can be effectively integrated into clinical practice to optimize adherence and therapeutic outcomes (Fig. 1). The perceived burden of AR/C and asthma extends beyond physical symptoms, profoundly affecting the patients’ QoL, emotional well being, daily functioning, and social participation, underscoring the need for patient-centered approaches in both disease management and outcome assessment.

Multiple studies have demonstrated that poorer control and greater severity of asthma and AR are strongly associated with reduced QoL [96–100]. Importantly, the differences in QoL between the good and partial control are modest, whereas the gap between the partial and poor control is substantial [101]. The overall burden of asthma and AR/C on QoL is often underestimated, with utility values for poorly controlled disease comparable to those seen in chronic conditions, such as type 2 diabetes mellitus, early-stage heart failure, and low disease activity rheumatoid arthritis [102–104]. These findings underscore the importance of the systematic assessment and proactive management of allergic respiratory diseases, particularly given the frequent underestimation of AR in clinical practice.

Patient adherence to AIT is a complex, multifaceted issue influenced by factors such as age, physician engagement, perceived treatment efficacy, convenience, patient education, and financial considerations [25]. Adherence can be defined as the extent to which a patient's behavior aligns with medical recommendations, involves initiation, implementation, and persistence [105]. Suboptimal adherence markedly reduces the therapeutic benefit and increases morbidity, hospitalization, and healthcare costs [24,106,107]. Age-related patterns show higher adherence in children, due to caregiver involvement, and in older adults, are likely linked to fewer competing obligations [108,109]. Physician-related factors are critical; regular follow-ups and a strong patient–doctor relationship significantly enhance adherence, with higher rates reported under the supervision of primary care physicians or pediatricians compared with specialists [107,110]. Perceived efficacy and patient expectations heavily shape adherence, particularly during the first year of treatment [111]. Unrealistic expectations or limited early improvement often lead to discontinuation [25]. Inconvenience, including treatment duration, administration frequency, perceived or occurred adverse effects, and logistical barriers, also impacts persistence, especially in SCIT and SLIT [112]. Better patient information regarding the expected nature and management of adverse reactions can improve adherence [113].

Patient education remains pivotal; comprehensive, tailored information on AIT's benefits, risks, and duration improves both understanding and compliance. Involving patients in therapeutic decisions, including the choice between SCIT and SLIT, enhances adherence, as does integrating adherence strategies from chronic disease management, such as telemedicine and structured follow-up, as highlighted in a survey conducted by Ballakur *et al.*, in which given the consistently high satisfaction reported across different AIT modalities, offering patients a choice between SLIT and SCIT reduced treatment attrition and improve long-term adherence [114].

Financial barriers and socioeconomic status influence adherence, with limited reimbursement and lower income associated with higher dropout rates. Regional disparities in healthcare coverage further complicate this issue as highlighted in a survey conducted by the national patient association “Respiriamo Insieme-APS” [19]. The association, founded in 2014 and registered in “Registro Unico Nazionale del Terzo Settore”, has approximately 3,500 members, including patients with respiratory disorders, caregivers, family members, and specialists such as allergists, anthropologists, pediatricians, pulmonologists, and psychologists.

These factors must be accompanied by the training of healthcare professionals who are both scientifically competent in managing AIT, from diagnosis to prescription, and attentive to the patients’ individual needs and expectations. A survey conducted by Josse *et al.* highlighted that for a large majority of patients who are aware of AIT but have not started, the main reason is that their physician never suggested it as a therapeutic option [113]. Clinicians should be equipped to communicate clearly and directly with patients, ensuring a transparent discussion about the substantial evidence-based benefits of AIT, thus fostering trust, shared decision-making, and long-term adherence.

To improve adherence, a multifaceted patient-centered approach is essential, integrating education, individualized treatment planning, physician support, and healthcare system interventions.

## PATIENT-REPORTED OUTCOMES MEASURES IN AIT STUDIES

Patient-reported outcome measures (PROMs), defined as standardized patient-administered instruments that capture the subjective impact of a disease or treatment from the patient's perspective, have become indispensable tools for the evaluation and management of rhinitis and chronic rhinosinusitis (CRS) [115^▪^,^116^]. PROMs serve a pivotal role in the assessment of symptom burden, QoL, therapeutic efficacy, and disease control in AR/C [117,118]. Moreover, PROMs are used to guide clinical decisions, for research (e.g., clinical trials for new drug development), to quantify patient-perceived quality across services and between providers, to help determine patient choice, and for policy decisions in allocating healthcare resources [115^▪^,^118^]. PROMs have great utility for developing and managing patient-specific treatment plans during meaningful, informed, and shared decision-making conversations. When providing patient-centered care, the outcome measures should reflect what is important to the patient. A summary of all commonly validated PROMs for AR/C is presented in Table 1.

**Table 1:** Commonly used validated PROMs in allergic rhinitis with or without conjunctivitis (adapted by Dykewicz *et al.* [115^▪^])

PROM	Type of PROMs	Reference
NOSE	Symptom score	Stewart *et al.* [120] and Zicari *et al.* [121]
CQ5	Symptom score	Stull *et al.* [135]
CQ7	Symptom score	Valero *et al.* [136]
ACS	Combined symptom and medication score	Hafner *et al.* [137]
Allergy-CSMS	Combined symptom and medication score	Sousa-Pinto *et al.* [138]
RCAT	Disease control	Schatz *et al.* [125], Meltzer *et al.* [124], and Fernandes *et al.* [123]
CARAT10	Disease control	van der Leeuw *et al.* [139]
CARATKids	Disease control	Linhares *et al.* [140], Borrego *et al.* [141], Amaral *et al.* [142], Laucis *et al.* [96], and Emons *et al.* [143]
ARCT	Disease control	Demoly *et al.* [132], Wang *et al.* [144], and Zhu *et al.* [145]
RQLQ and pRQLQ	QoL	Leong *et al.* [146], Okuda *et al.* [147]
Mini-RQLQ	QoL	Yuksel *et al.* [126]
RAPP	QoL	Molinengo *et al.* [148] and Braido *et al.* [127]

ACS, Allergy-Control-SCORE; ARCT, Allergic Rhinitis Control Test; CARAT10, Control of Allergic Rhinitis and Asthma Test; CQ5, Congestion Quantifier 5-Item Screener; CQ7, Congestion Quantifier 7-Item Test; CSMS, Combined Symptom Medication Score; NOSE, Nasal Obstruction Symptom Evaluation; PROM, patient-reported outcome measure; pRQLQ, pediatric RQLQ; QoL, quality of life; RAPP, RhinAsthma Patient Perspective; RCAT, Rhinitis Control Assessment Test; RQLQ, Rhinitis Quality of Life Questionnaire.

While a range of validated and nonvalidated tools are currently employed, symptom scores remain less rigorously validated, compared with those assessing disease control and QoL [115^▪^,^119^]. Widely utilized instruments include the Total Nasal Symptom Score (TNSS), Nasal Obstruction Symptom Evaluation (NOSE), and visual analog scale (VAS), which offer rapid patient-reported appraisals of symptom severity [120,121]. Additionally, Combined Symptom-Medication Scores (CSMS), which integrate symptom burden with medication usage, are recommended as primary efficacy endpoints in AIT trials by both the European Medicines Agency and European Academy of Allergy and Clinical Immunology, although comprehensive validation remains limited [122].

In the context of disease control, extensively validated PROMs, such as the Rhinitis Control Assessment Test (RCAT) and the Control of Allergic Rhinitis and Asthma Test (CARAT) are routinely applied, providing reliable and multidimensional evaluations encompassing symptom stability, pharmacological adjustments, and interference of disease on daily functioning [100,123–125]. The CARAT has been validated for assessing control in patients with AR and/or asthma, being the only PROM assessing control in patients with both diagnoses [118].

Within the QoL domain, measurement tools are the most comprehensively validated, with the Rhinitis Quality of Life Questionnaire (RQLQ) and its abbreviated form, the mini-RQLQ, considered the gold standards in both clinical trials and routine care [100,126]. Additional instruments, such as the RhinAsthma Patient Perspective (RAPP), further contribute to capturing the broader patient-perceived burden of disease, including in individuals with comorbid respiratory conditions [127,128]. Although QoL PROMs may require more time to administer than disease control scores, they provide complementary insights by capturing the dimensions of the disease burden that are often overlooked.

Moreover, Allergic Rhinitis and its impact on Asthma (ARIA) has developed a taskforce to include PROMs as digital biomarkers that can be used for a variety of purposes, for example, directing provider directed and patient self-directed management, research, and population health. The incorporation of PROMs into digital health solutions has significantly advanced, with initiatives such as the ARIA-led MASK-air project facilitating the development and validation of digital biomarkers for AR and asthma [129–131]. These include daily recordings of the CSMS, CARAT, EQ-5D VAS, and RAPP, enabling continuous real-time monitoring of disease control, treatment adherence, and impact on work productivity. Emerging data support the validity, reliability, and responsiveness of these digital tools, reinforcing their utility in both clinical decision-making and translational research frameworks.

## ROLE OF PATIENT ASSOCIATIONS IN ALLERGEN IMMUNOTHERAPY

**FIGURE 2:**
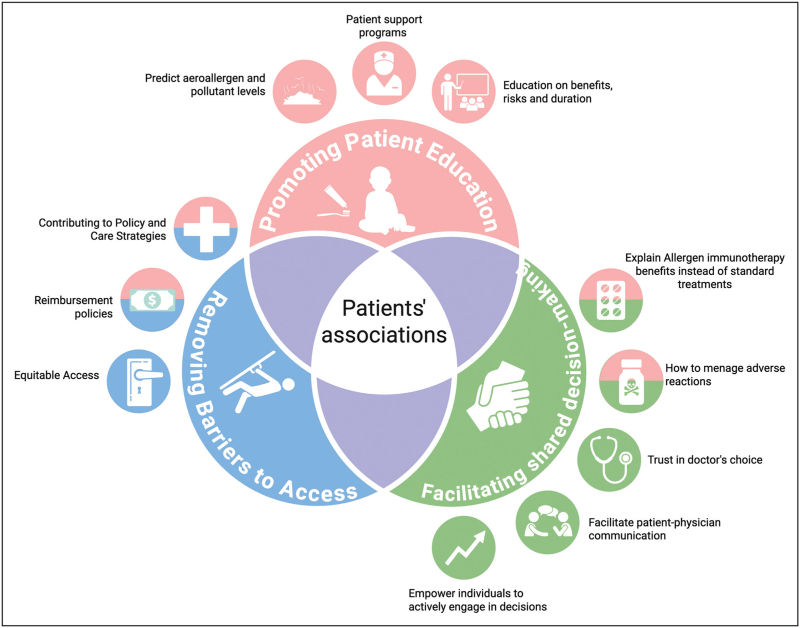
Multifaceted roles of patient associations in allergen immunotherapy. Created with BioRender.com.

Respiratory patient associations are becoming increasingly influential stakeholders in the management of allergic respiratory diseases, including AR and asthma [19,132]. These organizations play a pivotal role in promoting patient education, fostering health literacy, and empowering individuals to engage in decisions regarding their care actively (Fig. 2). By disseminating evidence-based information on disease management, treatment options, and the potential benefits and risks of interventions, such as AIT, patient associations contribute to improving adherence and facilitating shared decision-making. Furthermore, they serve as an essential interface between patients, healthcare professionals, and institutional stakeholders, advocating patient-centered care models and the removal of barriers to access, particularly in the context of AIT availability and reimbursement policies. Through organized advocacy efforts and public health campaigns, these groups promote awareness about allergic diseases and champion equitable access to effective therapies [132].

Moreover, patients’ associations have a crucial role in the so-called “patient support programs (PSPs)” that are intended to boost patient engagement, improve adherence (initiation, compliance, and persistence), help patients understand and manage their disease, improve QoL, and facilitate patient-physician communication, concordance, and trust [132]. In particular, in the field of AIT, PSPs have specific objectives, such as explaining the benefits of AIT and how it differs from other allergy treatments; increasing health literacy about allergic disease (from the diagnosis onwards, if possible); helping to understand and manage local or systemic adverse events and those related to the administration route; improving adherence year after year or season after season in order to obtain the disease-modifying benefits of AIT; and providing data on and/or predicting aeroallergen and pollutant levels on a local basis (for a patient's place of residence or when travelling), with customized alerts for specified allergen sources [132].

Notably, several European associations and international collaborations have demonstrated good practices in this domain, exemplifying how structured patient involvement can enhance clinical outcomes, support treatment adherence, and inform policy development [133,134]. Organizations raise awareness about specialized treatment centers and promote projects aimed at prevention, early diagnosis, and treatment, while actively campaigning against air pollution, smoking, and unhealthy lifestyle habits. They also run respiratory screening and awareness events and support patients in navigating hospital discharge and bureaucratic procedures to ensure community reintegration. Their participation in regional and national institutional meetings allows them to advocate for improved healthcare policies, bringing the patient's voice to policymaking, roundtables, and educational sessions [134].

## CHALLENGES AND FUTURE DIRECTIONS

Despite the growing recognition of AIT as a disease-modifying treatment, significant gaps remain in our understanding of patients’ perspective, particularly regarding their expectations, perceived barriers, and experiences throughout the therapeutic journey. To bridge these gaps, future research must prioritize co-designed studies in which patients are not only participants but also active partners in the development, implementation, and evaluation of care strategies. The integration of digital tools and telemedicine offers a promising avenue for enhancing patient education, streamlining follow-up, and ultimately improving adherence. Clinicians should be encouraged to adopt a more patient-centered approach, engaging in shared decision-making and addressing individual needs and preferences, while policymakers should work to ensure equitable access, updated reimbursement models, and support for real-life data collection. Only through such collaborative and innovative efforts we can optimize the impact of AIT and deliver care that is both evidence-based and truly responsive to patients’ lives.

## Acknowledgements

*None.*

### Financial support and sponsorship

*None.*

### Conflicts of interest

*M.G. reports personal fees from Sanofi, Thermo Fisher Scientific. E.H. reports fees for speaker activities and/or advisory boards participation from Sanofi, Regeneron, GSK, Novartis, AstraZeneca, Chiesi, Almirall, Bosch Healthcare, Lofarma, Orion Pharma, Celltrion-Healthcare, Apogee Therapeutics, Blueprint Medicines, Gentili, Firma outside the submitted work. GP reports fees for speaker activities and/or advisory boards participation from Lofarma, GSK, and AstraZeneca, outside the submitted work. The other authors declare that they have no conflict of interests to disclose in relation to this paper.*

## REFERENCES AND RECOMMENDED READING

Papers of particular interest, published within the annual period of review, have been highlighted as:

▪ of special interest
▪▪ of outstanding interest
